# *Daphnia* as a versatile model system in ecology and evolution

**DOI:** 10.1186/s13227-022-00199-0

**Published:** 2022-08-08

**Authors:** Dieter Ebert

**Affiliations:** Department of Environmental Sciences, Zoology, University of Basel, Vesalgasse 1, CH-4051 Basel, Switzerland

**Keywords:** *Daphnia magna*, *Daphnia pulex*, Cladocera, Branchiopoda, Cyclic parthenogenesis

## Abstract

Water fleas of the genus *Daphnia* have been a model system for hundreds of years and is among the best studied ecological model organisms to date. *Daphnia* are planktonic crustaceans with a cyclic parthenogenetic life-cycle. They have a nearly worldwide distribution, inhabiting standing fresh- and brackish water bodies, from small temporary pools to large lakes. Their predominantly asexual reproduction allows for the study of phenotypes excluding genetic variation, enabling us to separate genetic from non-genetic effects. *Daphnia* are often used in studies related to ecotoxicology, predator-induced defence, host–parasite interactions, phenotypic plasticity and, increasingly, in evolutionary genomics. The most commonly studied species are *Daphnia magna* and *D. pulex*, for which a rapidly increasing number of genetic and genomic tools are available. Here, I review current research topics, where the *Daphnia* model system plays a critical role.

## Natural habitat and life cycle

**Fig. 1:**
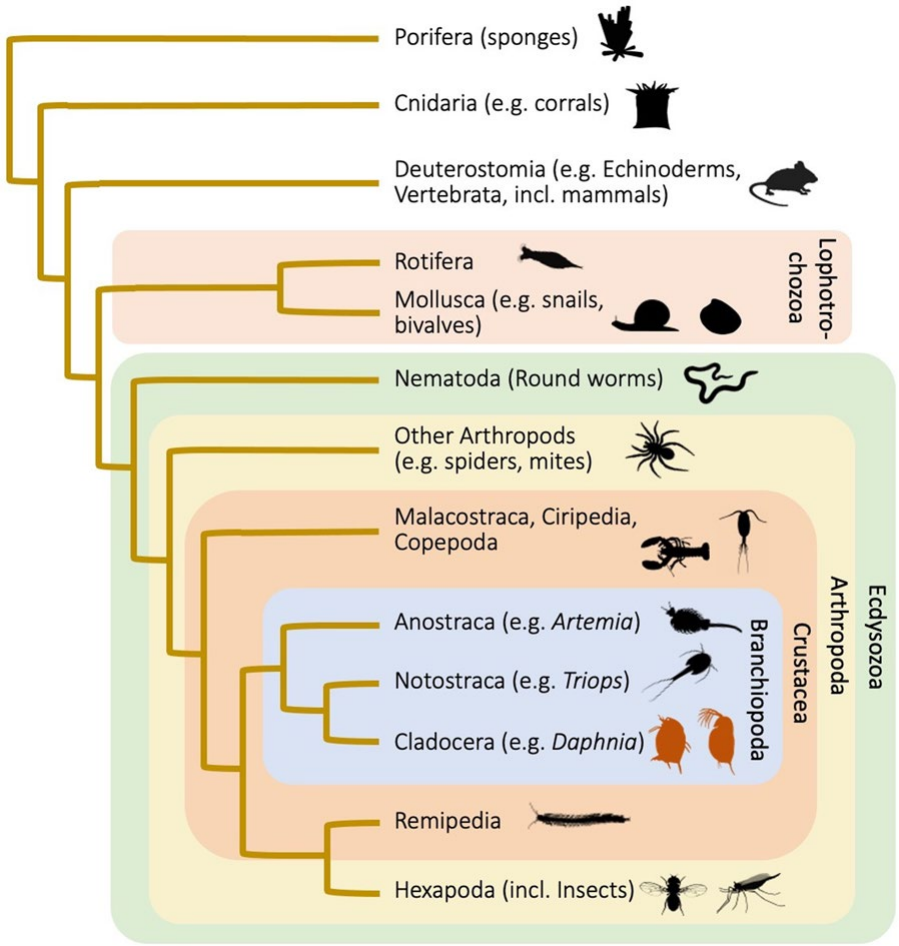
Schematic phylogenetic tree of the animals with a focus on *Daphnia* (in red). *Daphnia* are Cladocera, that form together with the Notostraca and Anostraca the Branchiopoda, formerly sometimes referred to as lower Crustaceans. Together with the Malacostraca, Copepoda, Cirripedia and some smaller taxa they form the Crustacea, now believed to be a paraphyletic taxon, because the Hexapoda are part of this clade, but not considered Crustaceans. Thus, *Daphnia* may be closer related to the model organism *Drosophila* than to a lobster. All of them are included in the Ecdysozoa, to which also the roundworms (Nematoda) belong. The tree was composed by taking various sources into account [24, 25, 127–130]. For animal pictograms see http://www.phylopic.org

**Fig. 2:**
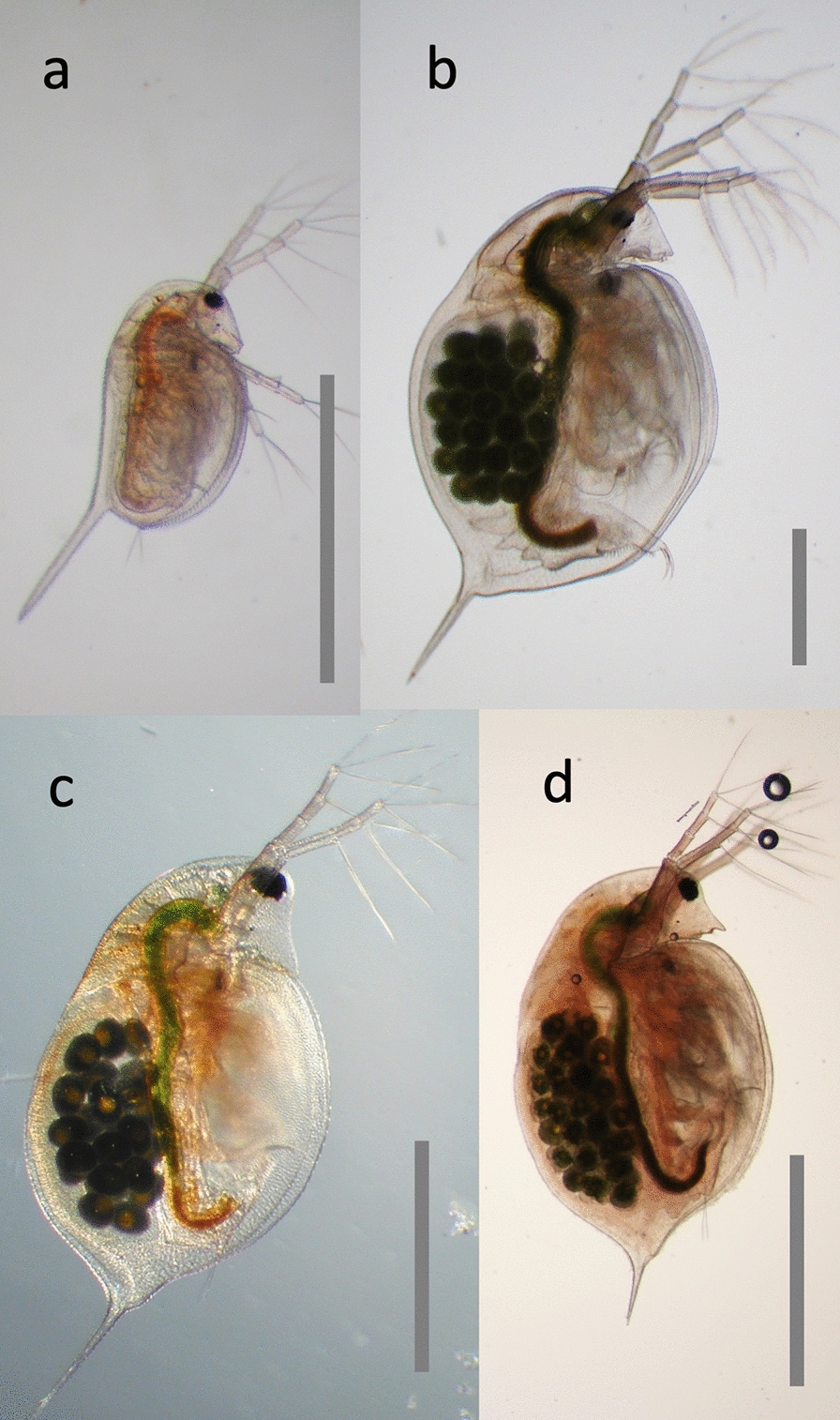
Three *Daphnia* species often used in biological research, representing three major clades of *Daphnia* within this large genus. **a** Newborn *D. magna*. **b** Adult *D. magna.* **c** Adult *D. longispina*. **d** Adult *D. pulex*. *D. magna* and *D. pulex* are predominately pond dwelling species, while *D. longispina* and related species are often found in large lakes. The three adult females carry parthenogenetic eggs (dark round objects) in their brood chambers. All animals are oriented with their head to the top and the ventral side to the right. The black round object in the head is the single complex eye. The large second antennae are used for swimming. All pictures by D. Ebert. Scale bar = 1 mm

**Fig. 3:**
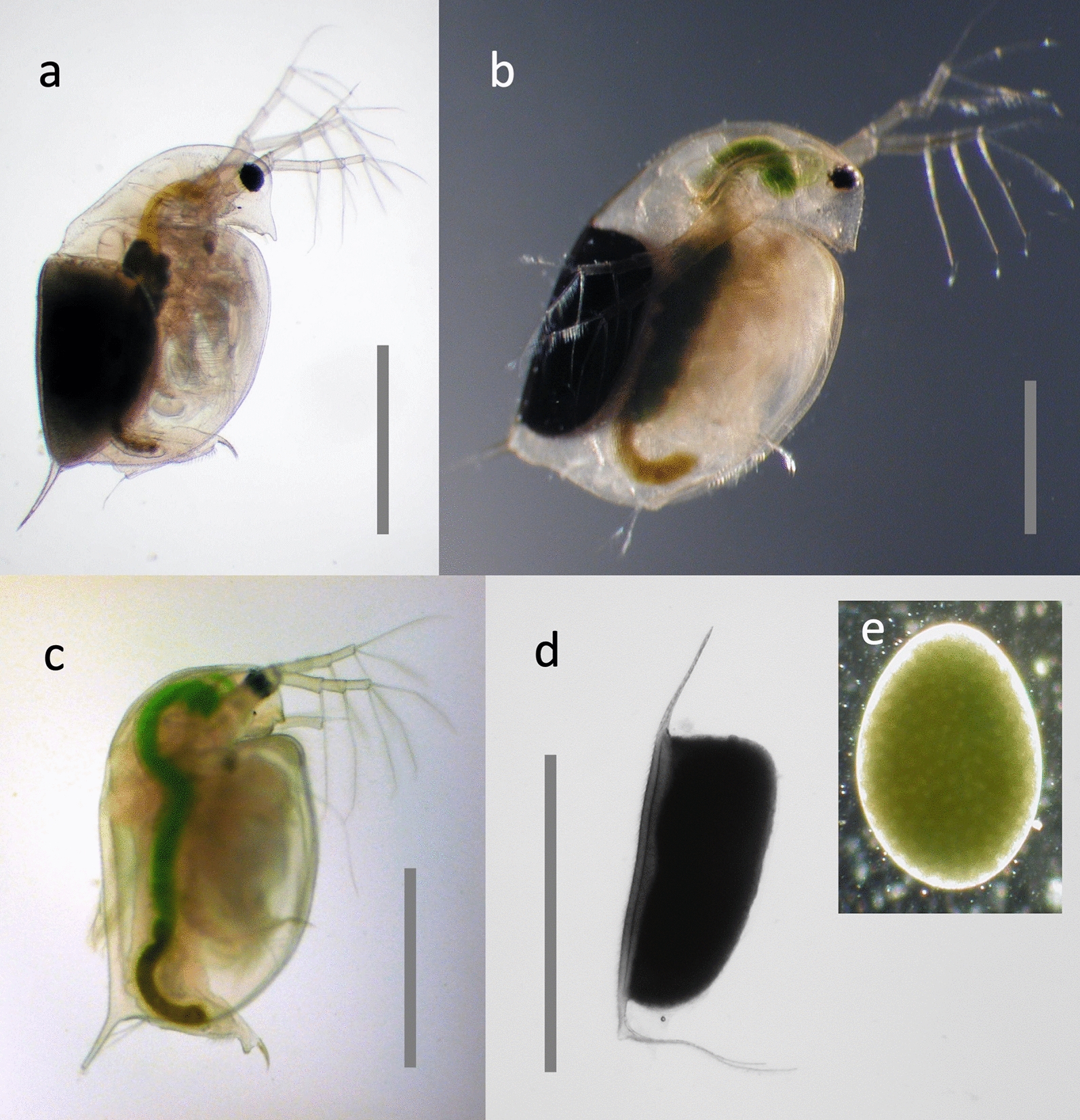
Sexual reproduction in *Daphnia*. **a** *D. pulex* with a sexual resting stage [= ephippium (plural: ephippia), the black structure]. **b** *D. magna* with resting stage. The dark mass in the centre part of the body (also visible in the animal in a) is the next asexual clutch being produced in the ovaries. **c** Male *D. magna*. **d** Freshly cast resting stage from *D. magna*, containing one or two embryos in developmental arrest. The resting stage has still parts of the female’s carapace attached to it (appendages in top and bottom), which is shed together with the resting stage. **e** Embryo from a resting stage. All pictures by D. Ebert. Scale bar = 1 mm

*Daphnia* is a genus of small planktonic crustaceans with a very wide geographic distribution. Its English name “water flea,” includes other members of the Cladocera order within the class Branchiopoda, united by the morphology of their trunk limbs (Fig. 1). There are over 100 described *Daphnia* species, each having a rather similar body architecture characterized by a relatively large head with one simple compound eye and a body encased in a bivalve-like shell (Fig. 2). *Daphnia* are more or less transparent so as to evade visually hunting predators, e.g., planktivorous fish. They moult four to six times before reaching maturity, but continue to moult and grow in regular intervals throughout their life. Newborn *Daphnia* (Fig. 2) resemble adults, except that they lack the well-developed dorsal brood pouch of adult females or the secondary sexual traits of males (Fig. 3) [1].

**Fig. 4:**
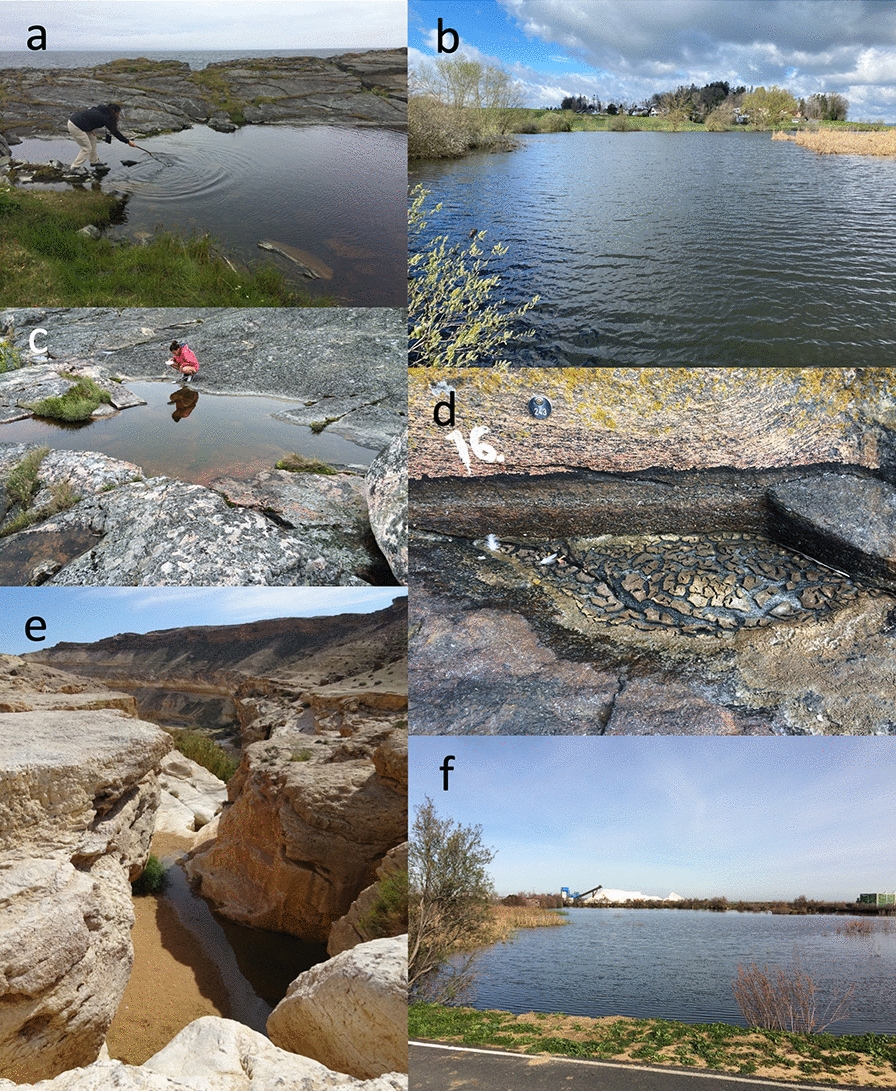
*Daphnia* occur in diverse fresh- and brackish-water habitats. **a** Northernmost located *D. magna* population so far reported, located on Vardo Island, Norway. **b** Permanent pond (Aegelsee) in Switzerland. **c** Shallow rock pool on the Island Granbusken, near Tvärminne in Southern Finland. **d** Dry rock pool close to the pool in picture c. The sediment surface is covered with *Daphnia* resting stages. **e** Temporary rain pond in the Negev Desert, Israel. **f** Salt water pond in Southern Spain. In the background piles of salt from a salt factory. All pictures by D. Ebert

*Daphnia* are often found in standing freshwater, from very small pools to very large lakes (Fig. 4), and although they may colonize salt water lakes or estuaries, they do not typically colonize sea water, as some species of related genera do. *Daphnia* are often a keystone species in ponds and lakes, where they are the main primary consumer, filter-feeding small suspended particles, in particular unicellular algae. As such, they play an important role in aquatic food webs, being themselves prey for fish or diverse invertebrate predators.

**Fig. 5:**
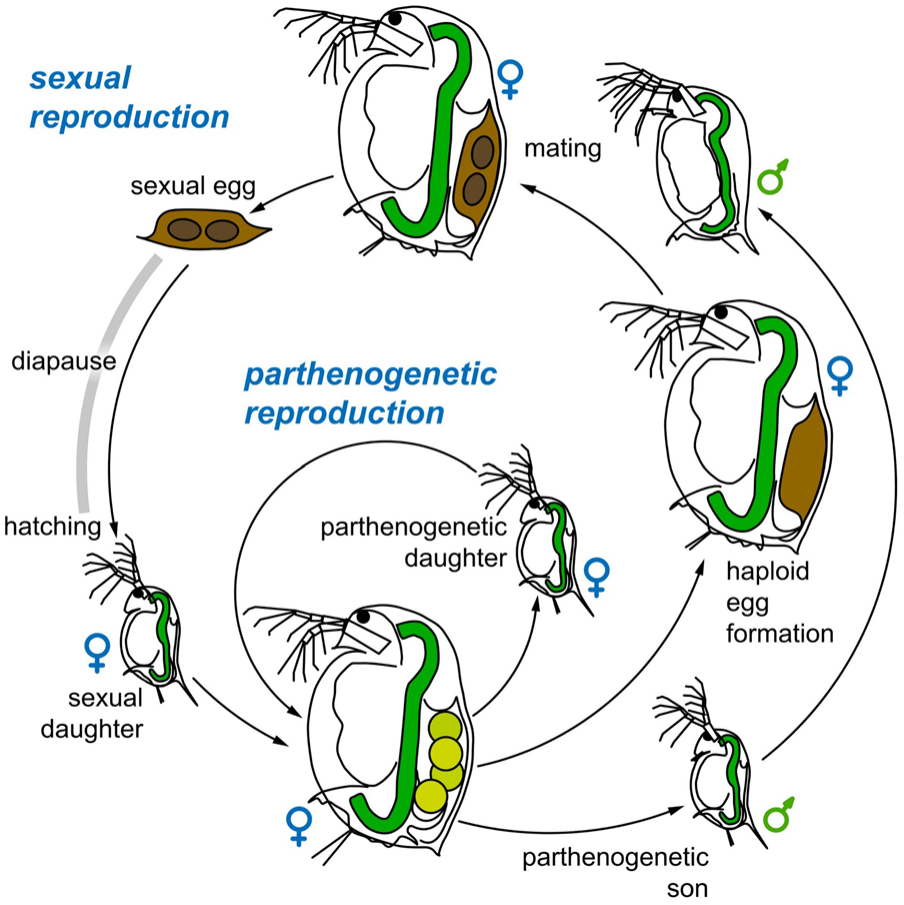
Life cycle of *Daphnia*. Adult females produce mostly asexual eggs, which develop directly inside their brood chamber (cycle: parthenogenetic reproduction). Mostly daughters hatch from these eggs. Occasionally, asexual eggs develop into males (sons). Some of the adult females in the population may switch to sexual reproduction and produce haploid eggs, which need fertilization by males (outer cycle: sexual reproduction). The female will eventually drop the fertilized eggs in an egg case made from her carapace (brown structure on top left; resting stage = ephippium). The ephippium will sink to the bottom of the water body, where it undergoes diapause. After diapause, one or two sexual offspring will hatch from it and develop into females. Conceptualization by D. Ebert and D. Vizoso. Drawing by D. Vizoso (available on Wikimedia)

*Daphnia* are well known for their ability to reproduce asexually (amictic parthenogenesis, i.e., diploid eggs capable of developing without fertilization) and under favourable conditions they can propagate asexually for many years (Fig. 5). In unstable environments and when environmental conditions deteriorate, *Daphnia* are able to switch to sexual reproduction. In this case populations produce first males (asexually!) and then haploid eggs that need fertilization. Production of sons and haploid eggs is regulated on the population level, with some genotypes never producing males (so called non-male-producers = NMP) or haploid eggs [2, 3]. Fertilized eggs start development, but then undergo developmental arrest. Resting embryos require a dormant period before they continue development, resulting in only female hatchlings (Fig. 5). The resting stage, also called ephippium (Fig. 3d), refers to the entire resting structure, i.e., the shell and the resting embryos they contain. Because the embryo is still encased in an egg shell and has an egg shape (Fig. 3e), it is often called resting egg, but this can be misleading. Some lines of *Daphnia* are known that are able to produce resting stages entirely asexually [4].

In the textbook description of the *Daphnia* life cycle, sexually produced offspring hatch from resting stages in the spring. These offspring then reproduce asexually across the season and finally terminate the planktonic phase by producing resting stages that endure the harsh winter conditions. However, studies in different habitats have revealed that this is the yearly life-cycle in temperate waterbodies common to Central Europe, where this model system was first developed. Depending on the species and habitat, any ecological aspect of this life-cycle may differ (Fig. 4) [5, 6]. For example, *Daphnia* may continue in the planktonic form throughout the winter or may persist in short-lived desert rain pools or tiny rock pools. Populations may only be active in winter (rainy season), produce no resting stages or produce resting stages asexually. The commonality among all *Daphnia* is that asexually reproducing animals are found in habitats, where water temperatures are at least for part of the planktonic season between about 10–30 °C and that they are able to outlive harsh conditions, such as temporary dryness, freezing and periods of low survival probability (e.g., predation, parasitism, toxic water conditions, such as anoxia) in the form of resting stages. Resting stages can survive many years in pond sediment (and laboratory fridges), as they tolerate drying and freezing.

## Field collections and laboratory culture

*Daphnia* can be collected from their natural habitats as either planktonic animals or in the form of their resting stages, which are often found in the sediments. Planktonic females can be cloned naturally, enabling researchers to produce unlimited numbers of clonal offspring and keep genetic lines for many generations under laboratory conditions. Clonal lines are kept in either natural fresh water, commercial mineral water or artificial medium (e.g., [7]) and are mostly fed unicellular green algae. On a commercial scale *Daphnia* are even farmed as fish food. Fifteen to 22 degrees is a good water temperature for asexual propagation for most species. Resting stages collected from pond and lake sediments or isolated from sliced and dated sediment cores can be hatched by exposure to oxygen-rich water under daylight and in ambient temperatures. Hatching success is, however, often low, especially for old resting stages, for reasons that we do not yet fully understand, although genetic effects (deleterious mutations) certainly play a role [8]. The oldest hatched resting stages was about 700 years old [9]. Each hatchling of sexually produced resting eggs is genetically unique and will start to reproduce clonally. Two hatchlings from one resting stage may be half or full-sibs [10].

Clonal reproduction allows us to produce genetically identical replicates so as to entirely control for genetic background. Sexual reproduction in the lab is more time consuming, because animals (usually small crowded populations) must first be induced to produce male offspring asexually, after which they produce haploid eggs for the males to fertilize [11]. The resulting eggs (actually developmentally arrested embryos) need an obligatory diapause of weeks to months, although methods have been developed to break diapause earlier [12]. Strong differences exist between species regarding the ease of sexual crosses in the laboratory, with *D. magna* currently being the most often-used species for routine crosses. It is possible to cross females with males from the same clone, resulting in genetically selfed offspring [11, 13]. This combination of sexual reproduction [outcrossing and genetic selfing (mother with clonal sons)] and clonal propagation allows researchers to conduct powerful genetic studies and create genetic panels that can be kept by clonal reproduction for many years [13, 14].

## Major interests and research questions

### Early beginnings

*Daphnia* research goes back several hundred years, with many important findings dating back to this old model system. To name just a few: August Weismann established his germ plasm theory working with water fleas [15], and Élie Metchnikoff [16] studied macrophages in *Daphnia* (Nobel prize 1908). The concept of phenotypic plasticity was also developed in relation to predator-induced defence in *Daphnia* [17], and the differentiation between prokaryotes and eukaryotes resulted from research on a *Daphnia* and its symbionts [18]. When advancements in modern biology began to be primarily driven by genetic research, *Daphnia* fell somewhat out of favour because of the difficulties in doing genetic crosses—a problem that has only recently been solved. Still, *Daphnia* has remained the prime model in ecological research and *Daphnia* is one of the best studied organism with regard to their ecology, with past or current focal areas are diel vertical migration [19], resurrection ecology [20], host—parasite interactions [21], community ecology [22] and climate change ecology [6, 23]. Moreover, in the last 20 years as genomics and genetics become incorporated into the tool box of *Daphnia* research (primarily *D. magna* and *D. pulex*), this model system is rising in prominence in the field of environmental genomics.

### Phylogenetic position

*Daphnia* are part of the Branchiopoda, the same group in which the tadpole shrimp *Triops* (Notostraca) and the brine shrimp *Artemia* (Anostraca) are placed (Fig. 1). Since the Crustacea are believed to a paraphyletic group (insects are phylogenetically part of the crustacea but typically not considered crustacea), the Branchiopoda are closer to insects than to other typical crustaceans, such as copepods, Malacostraca, Cirripedia and ostracods. Thus, *Daphnia* may be closer related to *Drosophila* than to lobster. Recent phylogenomic work has placed the genus *Daphnia* firmly in this phylogenetic tree and has clarified the evolution of the Cladocera [24] and the family of Daphniidae [25]. Crustacea are part of the Ecdysozoa, characterized by the need to moult their exoskeleton while growing (Fig. 1).

### Genome

The *Daphnia pulex* genome was the first crustacean genome to be sequenced [26, 27], followed by several other species [25, 28, 29]. Using flow cytometry, the genome size of several *Daphnia* species has been estimated to be about 230 MB, but genome assemblies are currently much smaller, suggesting that about 25% of the genome—likely the centromeric regions—is yet to be discovered. Several genetic maps have been published, allowing contigs to be sorted and oriented into chromosomes [30–32]. *Daphnia* have 10–12 chromosomes, but polyploid species exist as well.

A major surprise from the first genome was the large number of genes, with early counts around 30,000 [26]. However, a later re-assessment reduced this number to about 18,500 [28]. As both these studies used house-made unpublished bioinformatics scripts, it is difficult to understand the source of this difference and determine the better estimate, although unpublished data from *D. magna* (P.D. Fields & D. Ebert, unpublished) suggest that the higher estimate is closer to the real number.

### Local adaptation

With their habitat limited to standing water bodies, *Daphnia* populations are strongly subdivided. Gene flow is usually moderate, allowing populations to evolve rapidly in line with local environmental conditions. Animals collected from numerous waterbodies have had their genotypes preserved by clonal culture and then tested in common garden experiments (i.e., all animals being raised and phenotyped under the same environmental conditions) or reciprocal transplant experiments (i.e., transplantation of animals among environments, to work out the conditions to which animals are best adapted to). When genotypes demonstrate superior performance under the environmental conditions the animals have evolved in, it is considered evidence of local adaptation. *Daphnia* species have been shown to adapt locally to a long and growing list of environmental factors, including heavy metal pollution, predators, high temperature, habitat stability, photoperiod, water salinity, UV light [5, 33–39].

**Fig. 6:**
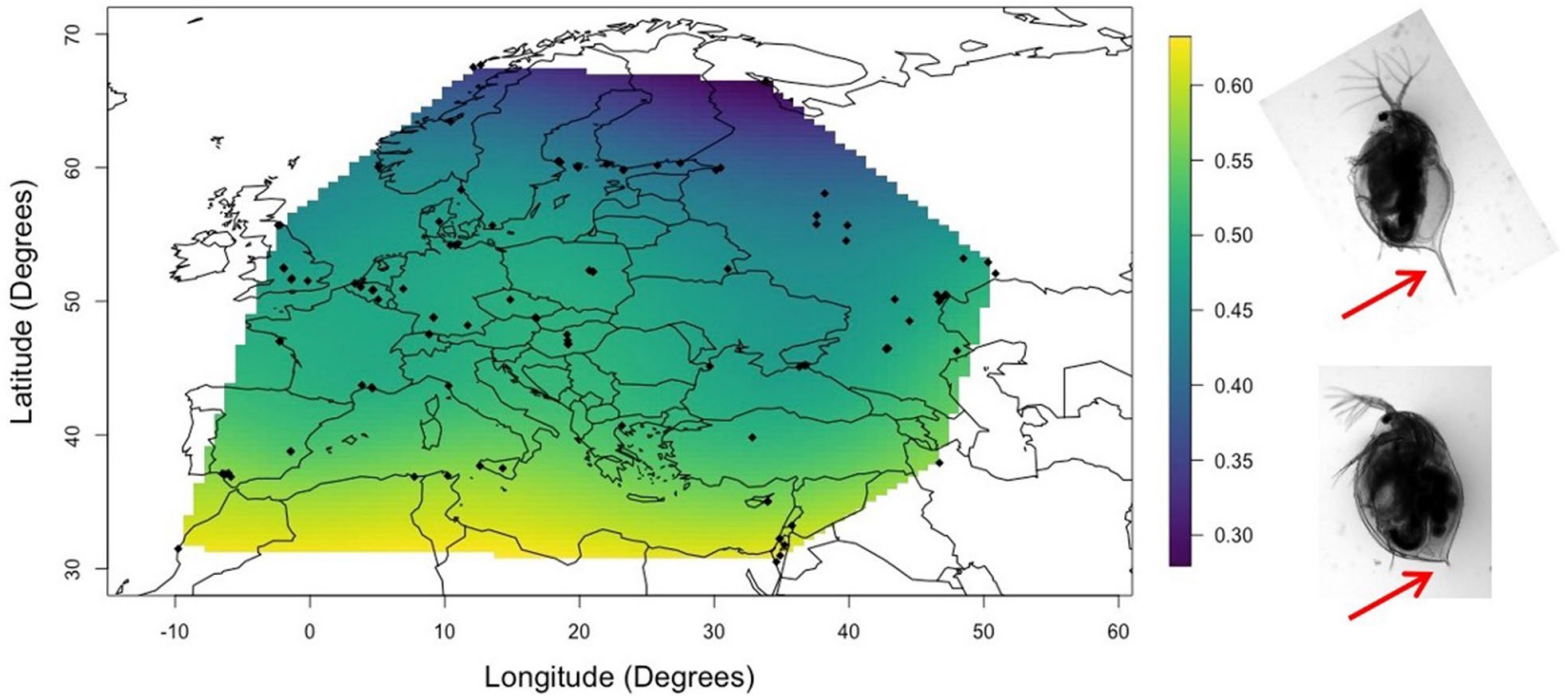
Example for geographic variation in a phenotypic trait: spina length at maturity across populations of *D. magna* (indicated as black dots). Phenotypes were generated in a common garden experiment and superimposed on a map according to the site of origin of the *Daphnia* clone. Scale on the right is in mm. On the right are examples of *D. magna* with short and long spina (red arrows). All genotypes are part of the *Daphnia magna* diversity panel a standing collection of hundreds of genotypes each from a different population

Although most local adaptation studies are based on comparing a few populations with multiple genotypes per population, a novel approach has been to collect single clones from as many populations as possible, instead of collecting multiple genotypes from each of a few populations. This method allows us to map phenotypes on geographic maps to investigate the influence of geography and climatic conditions (see Fig. 6 for an example how the length of the spina maps across Europe). It assumes, however, that a single genotype is representative of the entire population. The *Daphnia magna Diversity Panel* is a standing collection of hundreds of genotypes, each from a different population used for such studies. Indeed, the strong signal of local adaptation observed across these single genotypes suggests that single genotypes are—at least to some degree—representative of entire populations [6, 33, 39].

### Temporal adaptation and resurrection ecology

The long-lasting resting stages of *Daphnia* provide an even more compelling demonstration of the high evolutionary potential of these populations. While the majority of resting stages usually hatch during favourable conditions for the planktonic phase, some eggs may remain unhatched, though viable. With time, sediment buries these eggs, archiving them in layered sediments of the past. The field of resurrection ecology avails itself of this archive by collecting sediment cores, slicing them into layers and hatching the embryos from resting stages from the different layers [40, 41]. Information about the age of these layers may be obtained using radio isotope analysis. The resurrected *Daphnia* can then be tested in common garden experiments in response to different environmental variables, preferably those for which a record of past dynamics in the waterbody is present. These studies have revealed the rapid adaptive evolution of different *Daphnia* species to diverse environmental factors, such as density of predatory fish [42], heavy metal pollution [40], increased density of toxic cyanobacteria [43], and temporal dynamics in eutrophication [20]. One study resurrected, along with *D. magna*, the bacterial parasite *Pasteuria ramosa*, testing and confirming the idea that, over time, hosts and parasites coevolve [44]. Once they are combined with genomic methods, resurrection studies will be able to help us understand genetic targets of selection [45], e.g., by pool-sequencing of embryos from resting stages from different layers and studying the change of alleles (SNP-variants) over time.

### Host–parasite interaction

**Fig. 7:**
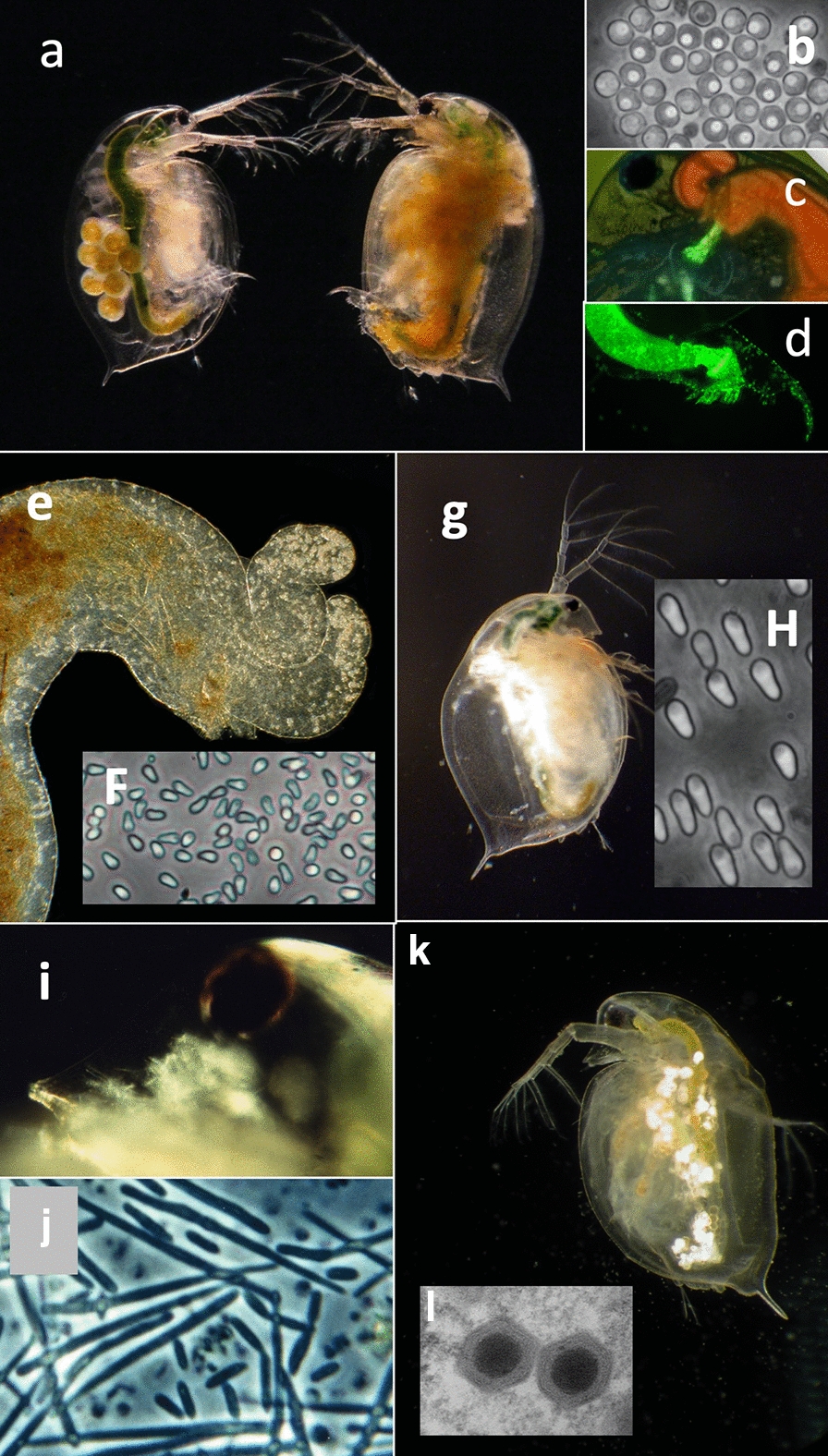
Examples of frequently studied parasites of *Daphnia*. **a–d** Bacterium *Pasteuria ramosa* colonizes the body cavity of the host. **a** Infected (right) and uninfected (left) *D. magna*. **b** Transmission stages (= spores) of *P. ramosa*. **c, d** Attachment of green fluorescent labelled *P. ramosa* spores to the oesophagus (**c**) and hindgut (**d**) of the host. Attachment of spores is required for the subsequent infection of the host [72, 131]. **e** Upper midgut of *D. magna* with spore clusters of the microsporidium *Ordospora colligata* in the appendices (upper right corner). The parasite colonized the gut epithelium of the host. **f** Spores of *O. colligata*. **g** *D. magna* infected with the microsporidian *Hamiltosporidium tvaerminnensis*. The parasite colonized the ovaries and fat body of the host. **h** Spores of *H. tvaerminnensis*. **i** Head of *D. magna* infected with *Metschnikowia bicuspidata*. The needles-like spores are visible through the transparent cuticle. **j** Spores of *M. bicuspidata*. **k** *Daphnia pulex* infected with the *Daphnia Iridovirus* (DIV-1), the causative agent of White Fat Cell Disease [68]. **l** Two DIV-1 particles. Picture taken by Jason Andras (**a**), David Duneau (**c**), Benjamin Hüssy (**d**), Patrick Mucklow (**g**) and Elena Toenshoff (**l**). All other pictures by Dieter Ebert

In their natural habitat, *Daphnia* are often infected at high prevalence with various parasites including viruses, bacteria, microsporidia, fungi, nematodes, cestodes and others (Fig. 7) [46, 47]. Many of these parasites can be co-cultured with their hosts, opening the door for experiments that examine host–parasite interactions on the individual and population levels. Compared to many other host–parasite systems, the *Daphnia*–microparasite system offers a level of experimental control that is unsurpassed, enabling us to test basic models of parasite ecology and evolution, such as the mass action principle [48], evolution of virulence [49], host and parasite evolution and coevolution [50, 51], the effects of ageing in host–parasite interactions [52, 53], infections with multiple parasites [54, 55] and parasitism in the face of predation [56] and other stressors [57]. An advantage of the *Daphnia* system that comes handy with regard to parasitism is the transparency of the host. Many infections can be diagnosed from outside, without killing the host (Fig. 7). Metchnikov’s [16] observation of phagocytosis was possible in vivo, by studying the fate of individual parasite cells in the living host.

The ability to use entire populations, furthermore, enables us to tackle questions that require replication on the population level, such as studies in experimental evolution [58] and epidemiology [59, 60]. Finally, the ease with which it is possible to study parasitism in natural populations allows us to compare data from natural population with data from laboratory experiments [51, 61] to understand the life cycles, epidemiology and evolution of several natural parasites of *Daphnia* [61].

While many parasites (and epibionts) of *Daphnia* have been used in field and lab-work, only a few have been studied intensively: the bacteria *Pasteuria ramosa* [62, 63], the microsporidia *Hamiltosporidium tvaerminnensis* [64] and *Ordospora colligata* [65, 66], the yeast *Metschnikowia bicuspidata* [21], the chitric fungus *Caullerya mesnilli* [67] and the Daphnia iridovirus DIV-1 [68]. Current research into the Daphnia parasites is focusing on coevolution and genetic epidemiology [51, 62], with new possibilities opening up as the genomes for most of these parasites becomes available. For example, a GWAS using different genotypes of *P. ramosa*, recently resulted in the first functional annotation of a parasite gene with regard to *Daphnia*–parasite interaction: a collagen-like protein was found to be responsible for the attachment of the bacteria to the host cuticle [69].

Cloning of *Daphnia* genotypes allows separating the effects of nature (genetic effects) and nurture (environmental effects) to a high degree of sophistication. This has been used to map genes involved in phenotypic traits, including resistance to parasites. Strong variation in host resistance has been reported [50, 50] for several parasites of *D. magna*. Studies that combine QTL-F2 panel mapping and GWAS have identified a number of regions and candidate genes in the *D. magna* genome that contribute to this variation, with strong variation in the underlying genetic architecture ranging, from single gene effects (major QTLs) to complex multigene effects (several minor QTLs), as they are typical for quantitative traits [71–73].

### Phenotypic plasticity

A hallmark of *Daphnia* biology is its phenotypic plasticity, defined as phenotypic variation expressed by the same genotype in response to environmental cues [74, 75]. Here again, the ability to clone genotypes of *Daphnia* has been instrumental in the development and research of this field. The most readily observable phenotypic plastic trait is the switch between asexual and sexual reproduction (Figs. 3, 5). This switch is triggered by deteriorating environmental conditions that lower the survival likelihood of asexual offspring and, therefore, make the production of sexually produced resting stages less costly [76]. A photoreceptor gene has been mapped in the genome of *D. magna* playing a crucial role for the switch from asexual to sexual reproduction [77].

**Fig. 8:**
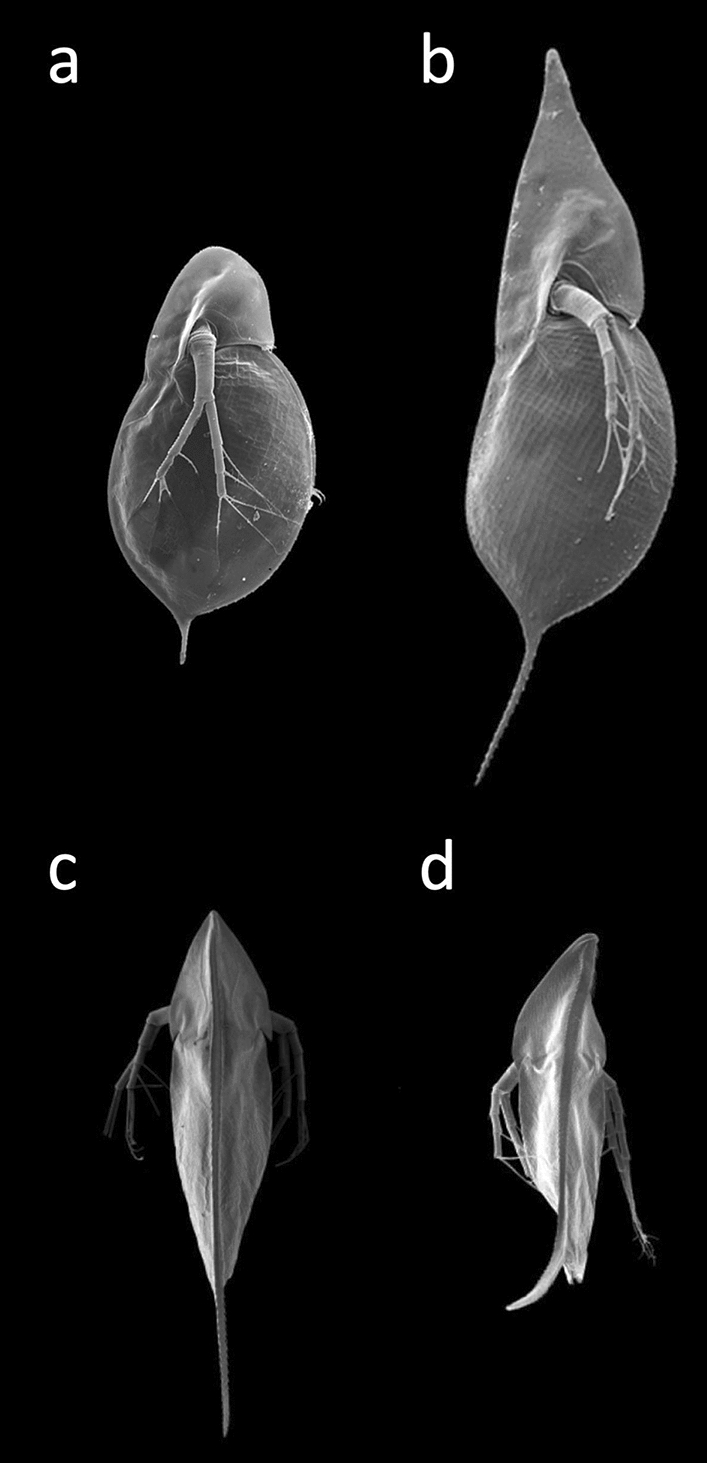
Waterfleas are able to react to cues from the environment, including water turbulences and infochemicals (kairomones) released by different predators, with the formation of highly specific structures, such as protective tail spines, helmets, and neck teeth. **a** *D. cucullata* in its normal (uninduced) phenotype (left) and **b** after induction (right). Helmets can be induced by water turbulence and by kairomones from fish. [81, 132]. **c** *D. barbata*: left control, **d** right induced by kairomones released by the predatory tadpole shrimp *Triops cancriformis*. The “twist”, a body torsion, induced by the kairomones reduces the likelihood of predation by *Triops* [75]. Pictures by Christian Laforsch, University of Bayreuth, Germany

With a well-developed set of gustatory receptors [78], *Daphnia* are able to sense chemical aspects of their environment, so called “infochemicals” or kairomones [79]. They indicate, for example, the presence of planktivorous fish, invertebrate predators or toxic blue–green algae, allowing the animals to respond in specific ways to reduce the threat (Fig. 8). They may change their body shape (e.g., develop helmets, neck–teeth, elongated tail spines), alter their life-history (e.g., size and age at maturity, offspring size) and change behaviour (phototaxis, swimming parameters) [80–82]. Adaptive phenotypic plasticity also drives responses to environmental conditions. For example, *Daphnia* produce fewer and larger offspring when food is scarce [83]; and animals produce specific haemoglobin variants tailored to the partial oxygen pressure of the water [84].

### Ecotoxicology

Without doubt, the field that spurs the most *Daphnia* publications is ecotoxicology, which examines the potentially toxic effects of chemical compounds on survival and reproduction. Many countries require that chemical compounds be tested with various organisms before they are produced, released or sold. *Daphnia* is one of these organisms, mostly *D. magna*, but also *D. pulex*, or, more rarely, other species. Guidelines by the Organisation for Economic Cooperation and Development (OECD) require tests, such as the Acute immobilisation test (a short-term or acute toxicity test) and the *Daphnia magna* Reproduction Test [85, 86]. These tests allow us to produce dose–response curves that can be used to determine parameters, such as the LC50 (lethal concentration at which 50 % animals die) and the lowest observed effect concentration (LOEC), parameters that help determine whether a chemical is potentially harmful for the environment and define any follow-up tests needed to understand the risk.

Moreover, the swimming behaviour of water fleas is increasingly used to quantify the effect of toxins, as well as to monitor the *Daphnia* response to changes in freshwater supplies for human consumption. Swimming speed, turn frequencies, acceleration and other parameters have all been observed to change as water quality changes. Using video tracking systems, these swimming-related parameters can be estimated in real time, and in the case of flow-through water surveillance systems, ensure that appropriate actions are taken. These systems are much faster than even the acute immobilization test and have become increasingly popular for research and applied aspects [87].

A rarely considered problem with using *Daphnia* in ecotoxicology is the genetic variation among *Daphnia* genotypes (clones). Different genotypes of the same species may differ strongly in the estimates of toxicity parameters [88]. Given the commercial interest in the production and sale of chemicals (incl. pharmaceuticals), this variation opens the door for biased reporting. So far, attempts to standardize the genotypes used across the world have not yielded a consensus.

### Evolutionary developmental biology

The discovery of the germ plasm by August Weismann in water fleas [15] was a major breakthrough at the time, but in the following hundred years *Daphnia* was not among the main players in the field of developmental biology. Today, *D. pulex* and *D. magna* are frequently used in the evo-devo field, often with a focus on comparative aspects [89], for example concerning the role of Hox genes [90–93], neuro development [94–96] and sex determination [97–100]. Along these lines diverse tool have been developed, for example an atlas for the staging of embryos [101] and diverse tools for genetic manipulation (see next section).

## Experimental approaches

### RNAi, CRISPR, TALEN

An expanding area of *Daphnia* research addresses the genetic mechanism underlying phenotypes, with the long-term goal of uncovering gene function in an environmental context. Manipulation of gene expression, as well as gene knock-in and knock-out technology are now possible for *Daphnia*, with microinjection-based RNA interference (RNAi) used routinely [102–104]. Clustered regularly interspaced short palindromic repeats/CRISPR-associated (CRISPR/Cas) system have been used for gene knock-outs [105, 106], but also for CRISPR/Cas-mediated knock-in via non-homologous end-joining, e.g., for reporter-genes [107]. Likewise, Transcription Activator-Like Effector Nucleases (TALENs) are increasingly used as a versatile genomic manipulation tool in *D. magna* [108–110]. These methods are primarily carried out via microinjection into the freshly laid asexual eggs of *Daphnia*, although other avenues such as bacterial feeding are being developed [111]. Mastering microinjection into the asexual eggs of *Daphnia* is still the main bottleneck for these methods, because the initially very fragile egg membrane breaks easily, but then hardens fast, so that injection is not possible anymore. The time window with the right conditions is short, even so a method has been develop to stretch it (collect eggs immediately after ovulation in ice-cold medium enriched with 80 mM sucrose) [102]. Another limiting factor is the relatively small clutch size of Daphnia, which in the lab is rarely larger than 30, even so in the field *D. magna* can produce clutches of more than 100 eggs. Thus, often eggs from multiple clutches need to be used. On the other hand, the combination of sexual and asexual reproduction is of great advantage when applying knock-in and knock-out methods. Manipulated genomes can be maintained in stable, clonal culture even in heterozygote state.

Current experimental approaches mainly focus on genes known from other organisms for diverse biological roles, such as development, sex expression (e.g., producing white eyes, knock-in of Green Fluorescent Protein) [97, 106, 107, 112–114]. So far, genes that have a function in the direct interaction of the *Daphnia* with their environment, for example genes for local adaptation, phenotypic plasticity, predator defence, parasite resistance, perception of environmental factors, such as water quality, photoperiod, and toxins, have hardly been manipulated [115], partly due to the low number of good candidates for genes with such function.

### QTL panels and GWAS

The first attempts to map phenotypes to genes in *Daphnia* was based on an F2 *D. magna* QTL panel. The position of two deleterious, but naturally segregating mutations were mapped [8]. The same standing QTL panel was later used to map the position of resistance genes to different parasites [71, 116] and genes related to sex induction and breaking of diapause [5, 117]. The mapping of phenotypes to genotypes has become much easier by the use of next generation sequencing for large number of genotypes, conducting genome wide association studies (GWAS). Examples include the mapping of a gene for sex induction, a non-male-producer gene and a parasite resistance super-gene [51, 77, 100].

### Transcriptomics

With phenotypic plasticity being a hallmark of *Daphnia* biology, it is an ideal system to study differential gene expression, as different phenotypes of the same genotype can be contrasted. This has been used intensively for diverse questions related to gene expression differences in different environments or treatments, such as responses to different predators, chemicals, environmental toxins, temperature, salinity, heavy metals, resistance to parasites, sex expression [118–121]. A database, dedicated to gene expression studies in Daphnia (http://www.daphnia-stressordb.uni-hamburg.de/dsdbstart.php) summaries the result of nearly 100 studies published until 2018 [118]. More than 50 % are from *D. magna*. Only seven studies of the entire data set analyzed whole-transcriptome expression profiles (using RNA-seq). Besides RNA-seq, proteomics is also rapidly developing for *Daphnia* as a powerful research tool [122].

### Karyotyping

*Daphnia* have rather small and condensed chromosomes, which posed severe difficulties on accurate karyotyping [123, 124]. However, new protocols allowed to move forward [125]. The number of chromosomes for *D. magna* is worked out to be 10 (*N* = 1) and is 12 for *D. pulex*

### Research community and resources.

The *Daphnia* community is rather large with a long tradition. A few hundred papers are published every year that feature *Daphnia* as the main study organism. The Cladocera community normally holds triannual symposia, but this schedule was interrupted due to the Coronavirus pandemic. A younger meeting series are the “*Daphnia* Genomics Consortium” meetings, which had convened only three times previously.

### Genomic resources

The first genome of *D. pulex* was already sequenced more than 10 years ago [26] and several genomes of other *Daphnia* species have since then be sequenced [25, 29] (https://www.ncbi.nlm.nih.gov/genome/?term=Daphnia). Genetic maps exist for *D. pulex* and *D. magna* [8, 30, 32, 126]. wFleaBase, the Daphnia Water Flea Genome Database (http://wfleabase.org/) gives an overview, but is not always up-to-date.

### Animal resources

Since clonal lines of *Daphnia* can very easily be produced by collecting females from natural populations and keeping them clonally in the laboratory, a tradition of widely used standard laboratory lines has not come about in the *Daphnia* community. Even in the field of ecotoxicology, several different genotypes are used by labs around the world. Some of the bigger *Daphnia* research groups have collection of clones from different species and population. One of the biggest collections is currently in the Ebert-research group at the University of Basel, with The Daphnia magna Diversity panel (fully sequenced clonal lines from more than 230 populations). The same lab also houses more than 300 F2 clones of a *D. magna* QTL panel [30] and more than 100 sequenced *D. magna* clones from one single population (the Swisspond panel) [51].

## Data Availability

Not applicable.
